# Interpretative phenomenological analysis and genetic counseling

**DOI:** 10.1002/jgc4.70061

**Published:** 2025-06-03

**Authors:** Rachel A. Starr, Jonathan A. Smith

**Affiliations:** ^1^ School of Psychological Sciences Birkbeck University of London London UK

**Keywords:** counseling, genetic health, genomic healthcare, health psychology, interpretative phenomenological analysis, lived experience, qualitative research, risk perception

## Abstract

The role of qualitative methods in healthcare research has gained acceptance over the last 30 years, and there are now a number of well‐established approaches available offering a diversity of aims, procedures, and epistemological emphases. This paper focuses on one such approach, Interpretative Phenomenological Analysis (IPA), to provide a discussion and critical overview of its current and future utility for genetic‐counseling research. First, the phenomenological and hermeneutic (interpretative) underpinnings of IPA, as well as its methodological commitments to inductive, idiographic, and interrogative working, are considered in the wider contexts of genetic and genomic science, and psychotherapy and counseling research. Then, research using IPA with more direct relevance for genetic counseling is considered in greater depth. This corpus includes those studies that speak to the most frequently endorsed activities of genetic counselors and pass quality evaluations specific to IPA. The literature is organized into three areas: (1) perceptions of genetic risk, (2) how genetic information, such as a diagnosis, personal test, or family history, is understood, and (3) the activity and experience of genetic counseling. Studies concerning perception of risk suggest it is experienced like presence on a radar, becoming more or less prominent or further or nearer from an individual's sense of themselves and their life. Studies concerning the understanding of genetic information imply the importance of multiple contexts in shaping understandings, but also that genetic information can shape experiences of other aspects of life and therefore that contexts may themselves be fluid. IPA used to consider experiences of genetic counseling suggests the importance of expertise in managing uncertainty and the centrality of sense‐making in the process for both counselor and counselee. Concluding remarks involve current and future compatibility between IPA and genetic counseling and the call for continuation of what is suggested to be a mutually beneficial dialogue between the two.

## 1 INTRODUCTION

The role of qualitative methods in healthcare and counseling‐related research has gained acceptance over the last 30 years and there are now a number of well‐established approaches available offering a diversity of aims, procedures, and epistemological emphases (Pitney et al., 2024). The differences between these and their implications for evidenced‐based research in healthcare are usefully outlined by Holloway and Galvin (2023), while a discussion of qualitative methodologies and their use in genetic‐counseling research is provided by Wainstein et al. (2023).^1^

Here we take the opportunity to focus on one such approach, Interpretative Phenomenological Analysis (IPA: Smith et al., 2022 where the approach is outlined in detail), to provide a discussion and critical overview of its current and future utility for genetic‐counseling research. To do so, we first briefly describe the theoretical commitments involved in doing IPA and their application in the contexts of psychotherapy and counseling and genomic medicine and healthcare, before turning specifically to genetic‐counseling and a detailed discussion of some select examples from the corpus.

## 2 WHAT IS INTERPRETATIVE PHENOMENOLOGICAL ANALYSIS?

IPA is a qualitative methodology wherein participants' subjective knowledge is foregrounded to develop understandings of a phenomenon from a first‐person perspective. It was originally articulated in the context of health psychology with an aim to maintain a rigorous experiential focus while remaining flexible and open to other methods and disciplines.

IPA is phenomenological in that researchers are concerned with experience on its own terms, resisting a priori, status quo, and personal preconceptions of a topic and choosing, rather, to focus, inductively, on *what it is like* for the experiencer. IPA is interpretative in two ways. First, experience itself is understood as inherently interpretative; things in the world are seen, encountered, and constituted as things, shaped by the meanings and structures such as culture and language that exist about them. Researchers using IPA therefore remain attentive to the ways participants make meaning as personally, bodily, socioculturally, and temporally situated beings. Second, in IPA, the researcher and research process are also considered interpretatively engaged. Researchers undertake the hermeneutic work of making sense of their participant's sense‐making. This involves attempting to understand a participant's perspective empathically, while remaining cognizant of its (and their own) situatedness.

In this sense, IPA is also interrogative, seeking after meaning that may not be obviously given on the surface of, for example, a description, but that can be drawn out through a tentatively questioning exploration of the meanings underlying what is said. Finally, IPA is idiographic, prioritizing detailed investigation of each individual participant's experience independently, before any attempts to consider similarities and differences at a group level are made.^2^

### 2.1 IPA in psychotherapy and counseling research

IPAs commitment to exploring experience idiographically lends itself to psychotherapy and counseling research where understandings from different positions such as practitioner or client, or before and after treatment, can be important. In an example of its flexible employment to this end, IPA has been successfully used in multiperspectival designs to explore counseling experiences of counselors and counselees (Larkin et al., 2018).

Practitioners such as Fragkiadaki et al. (2021) further suggest that the detailed inductive exploration of experience undertaken in IPA can benefit psychotherapy impact research by helping elucidate the often multi‐layered and hard to conceptualize experiences of therapeutic change or of specific therapeutic techniques or processes (see also Allan & Eatough, 2016). Additionally, the undertaking to consider experience in terms of what matters to each participant and to attend carefully to the ways they choose to express it has allowed researchers using IPA to gain insight into experiences of counseling and psychotherapy of those whose perspective may be particularly vulnerable or hard to access (for example Conroy et al., 2023; Farr et al., 2024). This renders it a potentially powerful tool in the context of genetic counseling where patients may face a range of complex physical, emotional, and social challenges to their engagements with both treatment and research.

### 2.2 IPA in genomic health research

Chapman and Smith (2002) described the contribution of IPA to what was then *the new genetics*, and subsequently, a sizeable corpus has developed. IPA has been used to tell us much about experiences of living with a genetic condition, from the perspectives of patients, their families and the professionals supporting them (Brugallé et al., 2021; Glasscoe & Smith, 2011; Smith et al., 2006) and has been employed in a range of creative designs tailored to the needs of different participants and research questions. Nisbet et al. (2022) used IPA with interview‐dyads to look at experiences of disclosure in Turner Syndrome from the unique joint perspectives of adolescents and their parents/guardians. Netherton et al. (2023) conducted IPA with a photo‐elicitation method (for an overview see Kyololo et al., 2023), to explore young‐people's experiences of being diagnosed with apert syndrome, and Barter et al. (2017) used IPA with Foucauldian discourse analysis (see Willig, 2017), to consider how people with Down syndrome thought about genetic testing and concurrently explore the discourses which might shape their perspectives.

## 3 IPA AND GENETIC‐COUNSELING RESEARCH

Our paper (Starr & Smith, 2021) offers reflections on this wider corpus and the types of knowledge it has generated for issues related to genetic and genomic health. Here, we concentrate on research particular to genetic counseling. The following is not intended as an exhaustive or systematic literature review but rather a focused discussion of some select work in order to highlight what good quality IPA has to offer. We have concentrated on research using IPA that speaks to the most frequently endorsed activities of genetic counselors (Ormond et al., 2024),^3^ passes quality assessment as per the criteria for evaluating IPA described in Smith (2011), and contains some reference to implications for genetic counseling. These articles organically constellate into three areas: (1) perceptions of genetic risk, (2) how genetic information, such as a diagnosis, personal test, or family history, is understood, and (3) the activity and experience of genetic counseling.

### 3.1 Perceptions of genetic risk

Risk perception has come to represent a substantial topic in genetic and genomic research including that undertaken using IPA (Etchegary & Fowler, 2008; Senior et al., 2002; Smith et al., 2002). Much of this work was discussed in Starr and Smith (2021), so here we focus on more recent publications. We take the opportunity to draw attention to this work not only because of its recency, but because it demonstrates the application of high‐quality IPA and the types of findings it can produce.

Cooper et al. (2024), explored the experience of living with known at‐risk status for Huntington's disease in the context of psychological wellbeing management. The study provides an example of the clear focus identified as necessary for good IPA in Smith (2011), considering specifically the experiences of individuals aware of their at‐risk status who had chosen not to pursue testing. The rationale is consistent with the phenomenological and interpretative principles of IPA. Extant research and understandings are drawn upon for steer without being presented as definitive and none are treated as substitute for exploration and curiosity. For example, although risk status is understood medically, guidance on psychological adjustment to this status is acknowledged to specify the experience of individuals aware of their risk for at least 12 months.

This focus facilitates detailed engagement with the experiences described, as reflected in the findings (Group Experiential Themes: GETs) developed *“you're constantly in limbo”: living in two worlds; “I have to live, just bloody live”: managing the possibility of a time‐limited lifespan; and “I try and try my hardest to look past the disease”: the exhausting quest to keep living well*, which evidence a second marker of quality in IPA: the analysis is interpretative, not just descriptive. The GETs speak to the meanings bound up in participant's accounts, rather than descriptions organized into topics. Risk here is experienced as a stuckness or limbo between two outcomes so divergent that they represent separate worlds. Living with this risk means ongoing effort to resist the existential weight of potentially limited lifespan, and the exhaustion of doing so despite the presence of the disease pushing itself into awareness.

Interpretative depth is further supported in the analytic commentary, which provides a third example of quality in IPA: it is carefully written. In this paper, attention to narrative detail and word choice help to convey a sense of the quality and texture of the experiences captured. For example, the theme concerning *looking* past the disease [our emphasis], is supported by delicate invocation of multiple forms of seeing. The authors communicate the way “unexpected observations” effect identity or bring the reality of HD into the fore, drawing on language that suggests visual occlusion (the expression “overshadowed by fear”). This is not merely a stylistic device, it echoes the participants' talk: Karen, for example, describes struggling to “picture” what her father had been like prior to developing HD symptoms. This subtle reflecting back enriches the narrative without overwhelming it, and acts like a tuning fork to amplify and clarify the interpretations offered.

The combination of a clear rigorous method and demonstration of interpretative depth and creativity provides useful responses to concerns that being interpretative or foregrounding subjective knowledge means abstracting away from real world meaning, application, or relevance. The themes are experientially resonant, tapping into something that feels authentic and intuitively human, allowing those who have not had the same experience to, nonetheless, get a sense of what it is like. Those wanting to understand or support someone in a similar position may well appreciate this kind of insight, as may individuals in similar circumstances facing decisions and wanting to explore their own thoughts and feelings.

Two other recent studies involving experiences of HD demonstrate similar qualities to Cooper et al. (2024). The focus in these is the experience of individuals who had tested positive for the HD gene expansion at least 1 year previously but had not experienced symptoms to the degree that would result in diagnosis of the condition. These individuals were not at risk in the sense that the presence of the mutation had been confirmed, and the research questions were not explicitly oriented toward risk; however, in each, findings revealed uncertainty, fear, and precarity, and of being encumbered by a sense of risk that weighed upon many aspects of everyday life.

Theed et al. (2018) looked specifically at participants expectations of psychological therapy. Findings revealed that anticipations of its worth were shaped by the way distress related to HD was made sense of. Depression was (by participants and those around them), often attributed to the neurological changes expected in HD. This not only impacted the perceived utility of therapy but was seen as indicating onset of the illness. However, the psychological impact of testing positive for HD was also acknowledged and attempts to untangle these attributions generated ambiguity as to what distress signified in terms of illness progression. Echoing the sentiment *“you're constantly in limbo”: living in two worlds* described by Cooper et al. (2024) this added a layer of uncertainty (is this a symptom?) to an already liminal position between the genetic and symptomatic presence of HD.

Notably, these participants described pursuit of genetic testing to escape the “limbo” (p. 211) of their at‐risk status. However, confirmation that they had the mutation generated a new purgatory, waiting for symptom onset. In the context of a changed rather than escaped risk, insight into the relative dominance of discourses surrounding distress in HD, particularly those involving its biological and hence inevitable and intractable basis, gains significance. Individuals hoping to escape the distressing position of not knowing found themselves countering a new stuckness, where distress had become the only position.

The experience of HD occurring between receipt of a positive test for the gene expansion and onset of symptoms, was the explicit focus of work by Wieringa et al. (2022) which asked “how do individuals experience the pre‐manifest stage of HD?” (p. 7). The findings again spoke to a sense of two worlds and how one might navigate space between them. The theme, *I have no intention of becoming symptomatic*, captured participants' accounts of distancing themselves from HD by thinking about it as something to be faced in the future. Until then, time was stalled, mentally separating these worlds as far as possible and in contrast with the other two studies, this limbo‐esque stasis seemed welcomed as participants attempted to pause movement between a present without HD and a future when it would become real.

Wieringa et al. (2022) also engaged with visual and foreground/background imagery and metaphor used by participants, exploring these to capture the felt presence of the condition even in its hidden pre‐manifest form: “HD became part of “my forever radar rather than my temporary radar” (Daisy) and “an unresolved threat just lurking in the background” (Angie)” (p. 12). This resonates with discussion in the other studies as to the degree to which HD could be looked past or around, and adds insight into the way thinking about time might impact this struggle.

Together these studies paint a picture of risk as the portent of an invisible presence coming into view, and suggest a complex relationship between the facticity and visibility of the condition, felt as presence on a radar. Significance was attributed to its invisibility, as an unknown to be feared or as a future to protect oneself from seeing. Movements to its detectable (as a test), senseable (as distress or other symptoms), or observable (by others) forms were each associated with their own new challenges and perceived conscriptions of what the world could be like.

Of course, findings of this type do not alleviate the physical, bodily constraints caused by HD or offer a way to remove the CAG repeat expansion in the huntingtin gene; the biological reality of HD remains, regardless of its visibility. However, in its lived form as a presence on a radar, it can be considered in terms of prominence, conspicuousness, and proximity, and these offer psychological pliability.

The role of different understandings in genetic health is considered further as we turn to the next area of research. However, it is important to stress at this point that we highlight different types of information, not to promote the superiority of one over another, but to make the case for the unique and valuable contribution of each.

### 3.2 Understanding genetic information

The picture of risk described clearly involves a very different type of information from that conveyed medically. Indeed, for the individuals involved, the information provided by their genetic code evoked multiple understandings, its meaning revisited, revised, and sometimes resisted.

The recognition that genetic information may be understood differently from different positions is unlikely to surprise those familiar with the genetic‐counseling profession. The role is often described as positioned at the interface between scientists, researchers, medical practitioners, and patients (Middleton et al., 2017; Quinn & Mazur, 2022), involving awareness of lay, scientific, and medical knowledges and the potential barriers between them (Davies et al., 2024; Van Der Giessen et al., 2021).

IPA is concerned with experience as embedded in a meaningful lifeworld; thus, it is sensitive to personal understandings and the details of their situatedness. The phenomenological undertaking, to resist preconceptions and instead follow what individuals identify as meaningful or important, allows researchers to uncover the intricacies of individual perspectives and tease out factors that may not have been anticipated or previously recognized. It can, therefore, be particularly useful to explore understandings arising within new or under‐explored circumstances such as those arising from developments in science and healthcare.

Bakur et al. (2022) for example, responded to the growing presence of genetic services in Arab countries, by investigating experiences of patients in cardio‐genetic research clinics in Saudi Arabia, diagnosed with autosomal dominant Long QT syndrome or who were carriers of Jervell and Lange Nielsen syndrome. The research aimed to explore the role of Islam in coping and decision‐making in order to consider implications for genetic counseling, which was also in higher demand. Findings revealed a complex relationship between religious beliefs and perspectives toward diagnosis. Participants described understanding their illness in terms of their religious beliefs, but these did not take the form of rigidly applied rules. For each participant, religious principles were personally interpreted as part of their efforts to make sense of their diagnosis. In themes describing *Interplay between belief in medicine and in religion*; and *the complex impact of diagnosis on religiosity*, the relationship was shown to be multidirectional. Religious beliefs shaped perspectives toward illness and medicine, and experience of illness shaped perspectives toward religion.

The role of contexts such as culture and religion in perceptions of genetic healthcare is well acknowledged (Zhong et al., 2021), but studies such as this highlight the way contexts may be fluid when understood as part of subjective experience and offer insight into the ways aspects of an individual's world such as their experience of culture or religion can be impacted by, as well as impact, understandings of genetic diagnosis and information. Findings of this kind may represent a useful resource for those hoping to support such patients, both by offering means to understand their thinking about genetic conditions and thus facilitate decision‐making and management, and to help understand the impact of genetic diagnoses on other aspects of their lives in order to support them psychologically.

Bakur et al. (2022) set out to consider understandings of genetic diagnosis in an under‐explored context. However, other contexts that are important in shaping understandings may be personal, hidden or particular to a rare condition. Here the inductive stance in IPA represents a powerful tool, allowing sensitivity to features that are hard to anticipate at study outset. For example, research by Geerts et al. (2017) considered experiences of hereditary hemorrhagic telangiectasia (HHT). Findings revealed the way symptoms had not prompted concern for individuals due to a particular context: their historic typicality within the family: “I always had nosebleeds, but my grandfather had nosebleeds, my mother had nosebleeds, it's not… […] It was entirely normal” (p. 614).

A deepened understanding of the way this normalcy shaped participants' experiences, is facilitated via the thematic arc presented and this demonstrates another indicator of quality in IPA: development of a compelling narrative. The first theme concerning normalization of symptoms is followed by the theme *From Emotional Shock to Upheaval After the Diagnosis*. Here, participants describe struggling to react to and understand their diagnosis, following the whiplash of discovering that their seemingly innocuous nosebleeds actually indicated a serious life‐long illness. The experiences detailed in these first two themes add weight and gravity to the final theme *Facing a Distressing Dilemma* where participants' decision‐making concerning testing their children becomes entangled with the personal upheaval and distress experienced during their own diagnosis. The authors suggest that the normalization demonstrated across this thematic arc has notable implications for counseling these patients, conferring barriers to seeking, receiving, and coming to terms with diagnosis and to subsequent health management and decision‐making.

When information such as a genetic diagnosis is at odds with personal understandings, be these of one's religion or body or health, it can cause instability and confusion. Conversely, diagnosis can act as a gravitational center, solidifying rather than disrupting sense‐making and (perhaps over?) the way genetic health is understood.

As part of a larger study to evaluate the impact of a new, more inclusive testing program (universal genetic testing in epithelial ovarian cancer), Shipman et al. (2017) looked at the way staff and patients thought about *BRCA1/BRCA2* testing following an ovarian cancer diagnosis. Staff saw the information associated with testing in two ways: as an extra burden for patients dealing with an emotionally demanding diagnosis, and as important knowledge that each had the right to consider. These two perspectives shaped their decisions regarding when and how to discuss testing with patients.

In contrast, patients indicated minimal concern with information about testing and what it could tell them, suggesting it felt inconsequential in contrast with their cancer diagnosis. The significance changed, however, when considering implications for relatives; patients described a sense of responsibility similar to that expressed by staff, to pass on knowledge despite it being potentially upsetting or unwanted. In this sense, both patients and staff expressed dual meanings of testing information—as potentially distressing and intrusive and as something of great importance.

In these studies, information concerning genetic health was understood in different, sometimes contradictory ways. Variation occurred between different individuals in different positions, and within the same person as their position changed, regardless of original source, be it symptoms, testing, or diagnosis. Nevertheless, as in the HD studies describing different forms of risk, in each case, understandings were underwritten by a sense of inescapable facticity. It was the persistent presence of nosebleeds that indicated both their normality and pathology, the feelings of inherent responsibility to pass on genetic information that overrode differing personal significances or potential to cause distress, the permanence of diagnosis inscribed genetically that gave religious belief malleability.

Such insights facilitate appreciation of different positions and may help efforts to determine appropriate communication and support. In addition, given the extent of the challenges associated with uncertainty present not only in the genetic‐counseling literature (Ballard et al., 2024; van der Schoot et al., 2024) but in health psychology more broadly (Rettie & Daniels, 2021; Schweizer et al., 2023; Zybarth et al., 2024), there may be added worth conveyed by the meaningful sureties uncovered. The fragments of certainty, uniquely present in the detail of a particular perspective or experience, offer a point of shared understanding from which other difficult discussions can proceed, or indeed further research can be developed.

### 3.3 Experiences of genetic counseling

The third body of research identified involves IPA used to explore how genetic counseling is experienced. Both patient and practitioner perspectives have been investigated, and topics have included conceptions of the role (Michie et al., 1999 before it was more clearly defined), the communication occurring within sessions (Smith et al., 2000), and the way practitioners make sense of patient's sense‐making in the context of genetic uncertainties (Aasen & Skolbekken, 2014).

MacLeod et al. (2002) explored patients perspectives toward what made genetic counseling effective. Participants described their appreciation of clear certain information, as expressed by Brian: “It was the first time I'd actually spoken to someone who knew actually what this disease were […] … So really when I did get there it was like explaining so far as it possibly could be explained.” (p. 150). Findings also revealed the reassurance offered by the expertise of the counselor, and the way this helped militate against upset in instances where clear‐cut yes/no information was not available, or when expectations for conclusive answers were met with uncertainty.

Spiers et al. (2020) also explored reflections on genetic counseling from patients' perspectives. Here IPA was used to evaluate a genetic‐counseling narrative group session for people who had tested positive for the Huntington's disease expansion. The challenge of uncertainty was present again here, but being part of the group allowed participants to gain new perspectives toward it. As described in the subtheme *Beneficial comparison with others living with the gene fault*, meeting people with the same condition offered a source of hope and reassurance. Participant Elaine, who had been very distressed by her test result, explained: “I was like looking at everyone in awe thinking wow, you know, they're all still here, they're still alive, they're still getting up in the morning.” The potency of possibility represented by other group members was further expressed by participant Graham: “one thing that stuck in my head was a woman who, who still doesn't have it, but she was diagnosed years ago. […] that's a big one for me, so I can just get on with my life” (p. 1019).

These findings point once again to the richness and detail of insight made possible through the focused idiographic examination undertaken in IPA. Furthermore, similar to findings concerning perceptions of risk and understanding of genetic information, the extracts from Brian (MacLeod et al., 2002) and Elaine and Graham (Spiers et al., 2020), disclose fragments of surety within unpredictable shifting territory; both suggest that the surety of expertise may lessen the grip of uncertainty. In the former, practitioner expertise carries assurance that something has been explained “so far as it possibly could be” giving a sense of abeyance. In the latter, others with the same condition offer expert testimony to what is possible so that despite the unknowns of when symptoms might develop one can remain “still alive”, “still getting up in the morning”, and able to “just get on with my life”.

As discussed in previous sections, the inductive commitment in IPA facilitates openness to discovery and can generate findings that fall outside the original research focus. Gilfillan and Carter (2023), conducted a study looking at Mayer Rokitansky‐Küster‐Hauser (MRKH) syndrome, a rare congenital disorder that is likely to have a genetic basis though the precise etiology has not yet been identified. They did not set out to explore counseling experiences, but identified aspects of living with the condition which bare relevance for genetic counseling nonetheless. Findings included themes describing struggles with identity, acceptance and shame. These were compounded in a final theme *The isolating impact of lack of medical professional knowledge*. In contrast with the expertise that reassured patients in the aforementioned studies, here lack of expert knowledge exacerbated difficulties, as captured by one participant's account of a (non‐genetic) counselor's excited response to the illness “Oh, that's really woooo” (p. 9) that enforced her sense of abnormality.

Attending to the experiential details conveyed by participants and allowing what is meaningful and significant to them to be foregrounded during the analysis can provide a rich source of information in sometimes unexpected ways. In this case, the findings arguably underline the need for practitioners with the specialist knowledge and skillset of genetic counselors, and combined with the other studies, make a strong case for the importance of their particular expertise. The work exploring MRKH provides a further reminder that experiential knowledge is one part of a bigger picture. Medical and scientific research to better understand the genetic basis of the condition is advocated for, with the hope of being able to offer patients better genetic counseling (Triantafyllidi et al., 2022).

## 4 IPA AND GENETIC‐COUNSELING RESEARCH—FUTURE CONTRIBUTIONS

As indicated by the changing terminology, human genetic and genomic science is a field notable for progression and expansion. We therefore revisit Chapman and Smith's (2002) discussion of what IPA can contribute to it, in the context of present‐day trends and developments.

IPAs fine‐grained engagement with personal experience provides opportunity for ingress into areas that are emerging, or little‐explored and so represents a powerful tool in the context of *a rapidly changing scientific and medical landscape*. Indeed, it is because genomic science continues to be a field of rapid advance, that researchers, clinicians and patients are regularly faced with novel issues generating the need to look beyond “off the shelf” responses and current understandings (Farsides & Lucassen, 2023, p. 2). Even the much‐discussed challenge of uncertainty continues to be newly actualized. Expansion of genetic testing generates new scope for results where significance is uncertain (Burke et al., 2022), and as knowledge advances, previously collected genetic data may offer new or different information with potential implications for patients whose results have effectively changed (Clift et al., 2020; Kaufman et al., 2023; Singer et al., 2019; Walsh et al., 2024).

This is no less a concern in genetic counseling. The profession has been described as one of the fastest growing in healthcare, both in number of practitioners and associated research base (Zierhut et al., 2024) granting the field similar momentum and dynamism. The emerging field of psychiatric genetic counseling, for example, suggests evolution of different positions toward the role and toward the genetic basis of psychological disorder and how it can usefully be understood by patients (Gatt‐Rutter et al., 2024). Similarly, genetic counselors continue to be presented with new scenarios and circumstances where they might be asked for support: those generated by the growing availability of direct‐to‐consumer genetic testing (Hsieh et al., 2021) or by increased interest in technological innovations such as use of chatbots to communicate test results or facilitate decision‐making (Ireland et al., 2021; Schmidlen et al., 2022). The sense of rapidly expanding demand is echoed in the emphasis on innovation in genetic‐counseling methods and service delivery models to meet the growing range of patient needs associated with advances in the tests, conditions, and treatments that are possible (Boothe et al., 2021; Scheinberg et al., 2021; Shickh et al., 2021).

This changing scientific and medical landscape has become associated with a *growing demand for perspectival knowledge* that includes diverse and novel viewpoints, to support new directions in healthcare (Horn & Merchant, 2024; Terry, 2020). In genetic and genomic health, personal experience is increasingly considered an invaluable resource (Kleiderman et al., 2024), particularly in areas where lay positions and expert discourses may differ in important ways (Nelson et al., 2024). In genetic counseling, there is great interest in understanding currently underrepresented populations (Young et al., 2021; Zakaria et al., 2023), and the positions of those concerned that their perspective is not only unheard but under threat (Madeo et al., 2011). IPA is ideally suited to respond to this demand. As reflected in the studies described, the phenomenological stance adopted in IPA, wherein existing a priori or assumed knowledges are resisted, facilitates engagement with personal experience in a non‐prescriptive fashion. This engagement is strengthened by the inductive commitment to foreground participants' perspectives over status quo, dominant or presumed understandings, facilitating sensitivity to overlooked positions and viewpoints.

A related trajectory in the development of genetic healthcare is the *mainstreaming of genomic science*, involving its increasing integration into clinical care across all stages of the lifespan (Rehm, 2017; Riess et al., 2024; Slomp et al., 2022; Snir et al., 2021). This has generated new questions concerning the way scientific understandings are translated into other contexts, be these clinical, specialist, non‐specialist, or lay perspectives and the potential issues this might provoke (Marshall et al., 2024; Nisselle et al., 2024). IPAs concern with knowledge*s*, as imparted in the studies concerning understandings of genetic information, and its commitment to the exploration of participants perspectives and experiences on their own terms, makes it particularly appropriate to explore issues arising from the integration and transmission of genomic science in new and different circumstances.

The role of individually tailored medicine and recognition that different people will have different needs and responses, within broader health infrastructures, has been identified as crucial to the future of healthcare (NHS England, 2024) and the goal to bring “genomic technologies closer to the patient”, spearheaded as central to its successful provision (UK Government, 2022). Following this *move toward personalized medicine*, genetic and genomic healthcare has increasingly involved a tension between prioritizing the individual and personal, and attending to the broader structures and contexts shaping their healthcare interactions. The need to involve patients and families in a way that is meaningful for them, in the context of large‐scale health‐equitable services is ever prescient (Marshall et al., 2024). At the same time it is recognized that the healthcare systems into which genomic technology is integrated are as complex, diverse and rapidly changing as the science itself, resulting in significant challenges to the delivery of genomic services (Long et al., 2021; Sperber et al., 2017).

IPA is unique in its idiographic commitment, thus offering special utility to speak to this tension. Taking an idiographic focus does not mean disconnecting the individual from the wider context of their life, culture, and everyday world. Rather, it involves paying careful attention to situatedness as part of idiographic investigation and a focus on each individual case independently before any tentative suggestions are made at a group level, thus allowing the nuances of individual experience and its context to be uncovered.

The ethos of flexibility and creativity central to IPA offers considerable scope for methodological innovation, allowing response to the variety of issues raised in genetics research. It also means that there is no instruction manual, which, if followed precisely, will generate good IPA, and while we clearly foster great ambitions for the use of IPA in genetic‐counseling research, we caution that overambition is a common pitfall for those new to the method.

The importance of training in IPA should not be overlooked, and we encourage seeking out appropriate guidance when learning the method. Resources have become more accessible with the growth in online learning, and the IPA website lists relevant groups, courses, and readings (IPA at BBK, 2024). In addition, we recommend avoiding complicated study designs until one is more experienced. Beginning with a modest project^4^ provides an opportunity to develop skills, confidence, and familiarity with working interpretatively and phenomenologically. There is much value in really getting to grips with the analysis of a single interview before attempting to wrestle multiple participant groups over multiple time points and a novel data collection method. And indeed, the knowledge generated from looking in depth at the experiences of a small group of people has great value on its own terms.

## 5 IPA AND GENETIC COUNSELING—SOME FINAL WORDS ON GOOD FIT

In addition to the congruities described between the methodology of IPA and the challenges associated with genomic science and healthcare, there is, arguably, shared ground when one considers the doing of genetic counseling and the doing of IPA.

Genetic counselors have described the work they undertake to make sense of their patient's sense‐making (Aasen & Skolbekken, 2014), echoing the double hermeneutic characterizing IPA wherein the researcher is trying to make sense of the participant who is trying to make sense of what is happening to them (Smith et al., 2022; Smith & Osborn, 2004).

Genetic counselors also describe the need to consider patient perspectives empathically while remaining cognizant that they are perspectives, navigating education and provision of medical information, within the supportive, historically nondirective ethos of counseling (Davies et al., 2024; Schupmann et al., 2020). This negotiation of positions resembles the ongoing movement made by researchers during the interpretative work of IPA, between hermeneutic positions of empathy and of suspicion; the former aiming to get as close as possible to the individual's experience, the latter probing it critically to draw out meanings that may be outside a participant's immediate access (Eatough & Smith, 2017; Smith et al., 2022).

High‐quality findings in IPA, as discussed, are generated by the interpretive commitment of researchers, brought to life by analytic engagement that is both creative and grounded in the data (Oliveri & Pravettoni, 2018). This involves a willingness to approach research material flexibly and engage with language imaginatively, probing metaphor and imagery to explore interpretations at different levels (Larkin & Thompson, 2011; Smith, 2004). Similarly, in genetic counseling, creative interactions and the use of visual imagery and linguistic metaphor have been described as offering many benefits. Such engagement may support communication of difficult or complex medical information to patients, or provide counselors a means to bridge understandings when a patient's thoughts or feelings are difficult to articulate or only partially formed (Sexton & James, 2022; Wagener, 2017).

Speaking to this compatibility, it is noted that genetic‐counseling research is often undertaken by practitioners and that research constitutes a requisite part of their training (Amendola et al., 2021). In this respect, although much of this paper describes the contributions IPA offers to genetic counseling, the valuable contributions of genetic counselors to research should not be overlooked. These extend far beyond undertaking research projects and involve the provision of their specialist expertise in a range of roles.

Genetic counselors can, for example, provide important direction concerning interpretation and dissemination of research results, and support participants during and after studies (Adžemović et al., 2024; Rutakumwa et al., 2020). Though these arguments are typically made in the context of medical research, the relevance for qualitative work, particularly that such as IPA, which can involve in‐depth discussion of sensitive, complicated, and potentially emotional issues, should not be understated. Relatedly, researchers may draw on their expertise to facilitate communication of information in a clinically accurate but not unduly directive fashion when considering topics that require medical delineation (e.g., risk status) or understanding (such as perspectives toward a new type of genetic test).

We hope, therefore, that this paper has made the case for what we see as the considerable complementarity between IPA and genetic counseling and that it prompts further dialogue between the disciplines. We suggest the benefits of such dialogue are bidirectional, and that researchers using IPA have much to gain from the expertise of genetic counselors and much to offer genetic‐counseling research through excellent application of IPA.

## AUTHOR CONTRIBUTIONS

Jonathan Smith and Rachel Starr contributed to the conception or design of the work and were responsible for the drafting the work or revising it critically for important intellectual content; final approval of the version to be published and; agree to be accountable for all aspects of the work in ensuring that questions related to the accuracy or integrity of any part of the work are appropriately investigated and resolved.

## FUNDING INFORMATION

No funding supported this article.

## CONFLICT OF INTEREST STATEMENT

We have no conflict of interest to declare.

## ETHICS STATEMENT

Human studies and informed consent: As a review article, this research did not involve collection of new data or human participants.

Animal studies: Not applicable.

## Footnotes

^1^
For further information about different methodologies and the practicalities of their application see Analyzing Qualitative Data in Psychology (Lyons & Cole, 2021) and Qualitative Psychology a Practical Guide to Research Methods (Smith, 2024).

^2^
Further discussion of the theoretical underpinnings of IPA can be found in Eatough and Smith (2017) and Larkin and Thompson (2011).

^3^

*Case preparation, Risk assessment and risk counseling, Educating about diagnosis and natural history, Educating about testing options, Educating about test results, and Contracting and establishing rapport* were indicated by over 90% of respondents.

^4^
Details of what an early study might look like, such as recommended sample sizes and example research questions are provided in Smith et al. (2022).

## References

[jgc470061-bib-0001] Aasen, T. , & Skolbekken, J.‐A. (2014). Preparing for and communicating uncertainty in cancer genetic counselling sessions in Norway: An interpretative phenomenological analysis. Health, Risk & Society, 16(4), 370–389. 10.1080/13698575.2014.927838

[jgc470061-bib-0002] Adžemović, T. , Kabbale, K. D. , Katagirya, E. , Mukisa, J. , & Wayengera, M. (2024). “A call to action”: The need for genetic counseling in Uganda. Genetics in Medicine Open, 2, 101879. 10.1016/j.gimo.2024.101879 39712973 PMC11658546

[jgc470061-bib-0003] Allan, R. , & Eatough, V. (2016). The use of interpretive phenomenological analysis in couple and family therapy research. The Family Journal, 24(4), 406–414. 10.1177/1066480716662652

[jgc470061-bib-0004] Amendola, L. M. , Golden‐Grant, K. , & Scollon, S. (2021). Scaling genetic counseling in the genomics era. Annual Review of Genomics and Human Genetics, 22(1), 339–355. 10.1146/annurev-genom-110320-121752 33722076

[jgc470061-bib-0005] Bakur, K. H. , Al‐Aama, J. Y. , Alhassnan, Z. N. , Brooks, H. , Clancy, T. , Manea, W. , Takroni, S. A. , & Ulph, F. (2022). Exploring the role of Islam on the lived experience of patients with Long QT syndrome in Saudi Arabia. Journal of Genetic Counseling, 31(4), 922–936. 10.1002/jgc4.1562 35194886

[jgc470061-bib-0006] Ballard, L. M. , Doheny, S. , Dimond, R. , Lucassen, A. M. , & Clarke, A. J. (2024). Predictive genetic testing for Huntington's disease: Exploring participant experiences of uncertainty and ambivalence between clinic appointments. Journal of Genetic Counseling, 34, e1911. 10.1002/jgc4.1911 38741209 PMC11735177

[jgc470061-bib-0007] Barter, B. , Hastings, R. P. , Williams, R. , & Huws, J. C. (2017). Perceptions and discourses relating to genetic testing: Interviews with people with down syndrome. Journal of Applied Research in Intellectual Disabilities, 30(2), 395–406. 10.1111/jar.12256 27168113

[jgc470061-bib-0008] Boothe, E. , Greenberg, S. , Delaney, C. L. , & Cohen, S. A. (2021). Genetic counseling service delivery models: A study of genetic counselors' interests, needs, and barriers to implementation. Journal of Genetic Counseling, 30(1), 283–292. 10.1002/jgc4.1319 32885542

[jgc470061-bib-0009] Brugallé, E. , Antoine, P. , Geerts, L. , Bellengier, L. , Manouvrier‐Hanu, S. , & Fantini‐Hauwel, C. (2021). Growing up with a rare genetic disease: An interpretative phenomenological analysis of living with Holt‐Oram syndrome. Disability and Rehabilitation, 43(16), 2304–2311. 10.1080/09638288.2019.1697763 31786957

[jgc470061-bib-0010] Burke, W. , Parens, E. , Chung, W. K. , Berger, S. M. , & Appelbaum, P. S. (2022). The challenge of genetic variants of uncertain clinical significance: A narrative review. Annals of Internal Medicine, 175(7), 994–1000. 10.7326/M21-4109 35436152 PMC10555957

[jgc470061-bib-0011] Chapman, E. , & Smith, J. A. (2002). Interpretative phenomenological analysis and the new genetics. Journal of Health Psychology, 7(2), 125–130. 10.1177/1359105302007002397 22114232

[jgc470061-bib-0012] Clift, K. , Macklin, S. , Halverson, C. , McCormick, J. B. , Abu Dabrh, A. M. , & Hines, S. (2020). Patients' views on variants of uncertain significance across indications. Journal of Community Genetics, 11(2), 139–145. 10.1007/s12687-019-00434-7 31432391 PMC7062975

[jgc470061-bib-0013] Conroy, D. , Smith, J. A. , Butler, S. , Byford, S. , Cottrell, D. , Kraam, A. , Fonagy, P. , Ellison, R. , Simes, E. , & Anokhina, A. (2023). The long‐term impact of multisystemic therapy: An experiential study of the adolescent‐young adult life transition. Journal of Adolescent Research, 38(5), 842–875. 10.1177/07435584211025323

[jgc470061-bib-0014] Cooper, H. , Simpson, J. , Dale, M. , & Eccles, F. J. R. (2024). Maintaining psychological well‐being when living at risk of Huntington's disease: An interpretative phenomenological analysis. Journal of Genetic Counseling, 34, e1965. 10.1002/jgc4.1965 39252438 PMC11953587

[jgc470061-bib-0015] Davies, R. , Price Tate, R. , & Taverner, N. V. (2024). What next for “counseling” in genetic counseling training: A co‐production workshop exploring how CBT and ACT approaches can contribute to the genetic counseling toolkit. Journal of Genetic Counseling, 33(1), 124–128. 10.1002/jgc4.1868 38379347

[jgc470061-bib-0016] Eatough, V. , & Smith, J. A. (2017). Interpretative phenomenological analysis. In C. Willig & W. Stainton‐Rogers (Eds.), The Sage handbook of qualitative research in psychology (2nd ed., pp. 193–211). Sage.

[jgc470061-bib-0017] Etchegary, H. , & Fowler, K. (2008). ‘They had the right to know.’ Genetic risk and perceptions of responsibility. Psychology & Health, 23(6), 707–727. 10.1080/14768320701235249 25160812

[jgc470061-bib-0018] Farr, J. , Rhodes, J. E. , Baruch, E. , & Smith, J. A. (2024). Early intervention in psychosis for first episode psychotic mania: The experience of people diagnosed with bipolar disorder. Journal of Mental Health, 33(4), 500–506. 10.1080/09638237.2024.2332805 38588707

[jgc470061-bib-0019] Farsides, B. , & Lucassen, A. M. (2023). Ethical preparedness and developments in genomic healthcare. Journal of Medical Ethics, 51, 213–218. 10.1136/jme-2022-108528 PMC11877103 37268409

[jgc470061-bib-0020] Fragkiadaki, E. , Triliva, S. , & Anagnostopoulos, F. (2021). Application of interpretative phenomenological analysis methodology in psychotherapy impact research: Experience of psychotherapy of a person with multiple sclerosis. Qualitative Methods in Psychology Bulletin, 31, 26. 10.53841/bpsqmip.2021.1.31.26

[jgc470061-bib-0021] Gatt‐Rutter, T. , Forrest, L. , Sexton, A. , & Isbister, J. (2024). Consumer attitudes and preferences toward psychiatric genetic counselling and educational resources: A scoping review. Patient Education and Counseling, 123, 108229. 10.1016/j.pec.2024.108229 38461792

[jgc470061-bib-0022] Geerts, L. , Fantini‐Hauwel, C. , Brugalle, E. , Boute, O. , Frenois, F. , Defrance, L. , Manouvrier‐Hanu, S. , Petit, F. , & Antoine, P. (2017). The subjective experience of patients diagnosed with hereditary hemorrhagic telangiectasia: A qualitative study. Journal of Genetic Counseling, 26(3), 612–619. 10.1007/s10897-016-0033-z 27796677

[jgc470061-bib-0023] Gilfillan, R. , & Carter, P. (2023). Issues of identity, perceptions and isolation: An interpretative phenomenological analysis of women's experience of Mayer‐Rokitansky‐Küster‐Hauser (MRKH) syndrome. Journal of Health Psychology, 29, 13591053231199253. 10.1177/13591053231199253 37771134

[jgc470061-bib-0024] Glasscoe, C. , & Smith, J. A. (2011). Unravelling complexities involved in parenting a child with cystic fibrosis: An interpretative phenomenological analysis. Clinical Child Psychology and Psychiatry, 16(2), 279–298. 10.1177/1359104510383207 21212082

[jgc470061-bib-0025] Holloway, I. , & Galvin, K. (2023). Qualitative research in nursing and healthcare. John Wiley & Sons.

[jgc470061-bib-0026] Horn, R. , & Merchant, J. (2024). Ethical and social implications of public–private partnerships in the context of genomic/big health data collection. European Journal of Human Genetics, 32(6), 736–741. 10.1038/s41431-024-01608-9 38627540 PMC11153602

[jgc470061-bib-0027] Hsieh, V. , Braid, T. , Gordon, E. , & Hercher, L. (2021). Direct‐to‐consumer genetic testing companies tell their customers to ‘see a genetic counselor’. How do genetic counselors feel about direct‐to‐consumer genetic testing? Journal of Genetic Counseling, 30(1), 191–197. 10.1002/jgc4.1310 32706156

[jgc470061-bib-0028] IPA at BBK . (2024). Interpretative phenomenological analysis at Birkbeck . https://www.ipa.bbk.ac.uk/

[jgc470061-bib-0029] Ireland, D. , Bradford, D. , Szepe, E. , Lynch, E. , Martyn, M. , Hansen, D. , & Gaff, C. (2021). Introducing Edna: A trainee chatbot designed to support communication about additional (secondary) genomic findings. Patient Education and Counseling, 104(4), 739–749. 10.1016/j.pec.2020.11.007 33234441

[jgc470061-bib-0030] Kaufman, R. , Timmermans, S. , & Raz, A. (2023). Genomic uncertainty and genetic counsellors' professional authority. Sociology of Health & Illness, 45(3), 485–502. 10.1111/1467-9566.13582 36424363

[jgc470061-bib-0031] Kleiderman, E. , Boardman, F. , Newson, A. J. , Laberge, A.‐M. , Knoppers, B. M. , & Ravitsky, V. (2024). Unpacking the notion of “serious” genetic conditions: Towards implementation in reproductive decision‐making? European Journal of Human Genetics, 33, 158–166. 10.1038/s41431-024-01681-0 39127803 PMC11840117

[jgc470061-bib-0032] Kyololo, O. M. , Stevens, B. J. , & Songok, J. (2023). Photo‐elicitation technique: Utility and challenges in clinical research. International Journal of Qualitative Methods, 22, 16094069231165714. 10.1177/16094069231165714

[jgc470061-bib-0033] Larkin, M. , Shaw, R. , & Flowers, P. (2018). Full article: Multiperspectival designs and processes in interpretative phenomenological analysis research. Qualitative Research in Psychology, 16(2), 182–198. 10.1080/14780887.2018.1540655

[jgc470061-bib-0034] Larkin, M. , & Thompson, A. R. (2011). Interpretative phenomenological analysis in mental health and psychotherapy research. In D. Harper & A. R. Thompson (Eds.), Qualitative research methods in mental health and psychotherapy: A guide for students and practitioners (pp. 101–116). John Wiley & Sons, Ltd. 10.1002/9781119973249

[jgc470061-bib-0035] Long, J. C. , Gul, H. , McPherson, E. , Best, S. , Augustsson, H. , Churruca, K. , Ellis, L. A. , & Braithwaite, J. (2021). A dynamic systems view of clinical genomics: A rich picture of the landscape in Australia using a complexity science lens. BMC Medical Genomics, 14(1), 63. 10.1186/s12920-021-00910-5 33639930 PMC7912922

[jgc470061-bib-0036] Lyons, E. , & Cole, A. (Eds.). (2021). Analysing qualitative data in psychology (3rd ed.). SAGE Publications Ltd. https://uk.sagepub.com/en‐gb/eur/analysing‐qualitative‐data‐in‐psychology/book268665

[jgc470061-bib-0037] MacLeod, R. , Craufurd, D. , & Booth, K. (2002). Patients' perceptions of what makes genetic counselling effective: An interpretative phenomenological analysis. Journal of Health Psychology, 7(2), 145–156. 10.1177/1359105302007002454 22114234

[jgc470061-bib-0038] Madeo, A. C. , Biesecker, B. B. , Brasington, C. , Erby, L. H. , & Peters, K. F. (2011). The relationship between the genetic counseling profession and the disability community: A commentary. American Journal of Medical Genetics Part A, 155(8), 1777–1785. 10.1002/ajmg.a.34054 PMC5240749 21567935

[jgc470061-bib-0039] Marshall, D. A. , Hua, N. , Buchanan, J. , Christensen, K. D. , Frederix, G. W. J. , Goranitis, I. , Ijzerman, M. , Jansen, J. P. , Lavelle, T. A. , Regier, D. A. , Smith, H. S. , Ungar, W. J. , Weymann, D. , Wordsworth, S. , & Phillips, K. A. (2024). Paving the path for implementation of clinical genomic sequencing globally: Are we ready? Health Affairs Scholar, 2(5), qxae053. 10.1093/haschl/qxae053 38783891 PMC11115369

[jgc470061-bib-0040] Michie, S. , Smith, J. A. , Heaversedge, J. , & Read, S. (1999). Genetic counseling: Clinical geneticists' views. Journal of Genetic Counseling, 8(5), 275–287. 10.1023/A:1022930215375 26142374

[jgc470061-bib-0041] Middleton, A. , Marks, P. , Bruce, A. , Protheroe‐Davies, L. K. , King, C. , Claber, O. , Houghton, C. , Giffney, C. , Macleod, R. , Dolling, C. , Kenwrick, S. , Scotcher, D. , Hall, G. , Patch, C. , & Boyes, L. (2017). The role of genetic counsellors in genomic healthcare in the United Kingdom: A statement by the Association of Genetic Nurses and Counsellors. European Journal of Human Genetics, 25(6), 659–661. 10.1038/ejhg.2017.28 28327572 PMC5518913

[jgc470061-bib-0042] Nelson, J. P. , Tomblin, D. C. , Barbera, A. , & Smallwood, M. (2024). The divide so wide: Public perspectives on the role of human genome editing in the US healthcare system. Public Understanding of Science, 33(2), 189–209. 10.1177/09636625231189955 37638525

[jgc470061-bib-0043] Netherton, J. , Horton, J. , Stock, N. M. , Shaw, R. , Noons, P. , & Evans, M. J. (2023). Psychological adjustment in Apert syndrome: Parent and young person perspectives. The Cleft Palate Craniofacial Journal, 60(4), 461–473. 10.1177/10556656211069817 34967688

[jgc470061-bib-0044] NHS England . (2024). NHS long term plan. Chapter 1: A new service model for the 21st century. NHS England. https://www.longtermplan.nhs.uk/online‐version/chapter‐1‐a‐new‐service‐model‐for‐the‐21st‐century/

[jgc470061-bib-0045] Nisbet, M. , O'Connor, R. , Mason, A. , & Hunter, E. (2022). A qualitative study utilizing interpretative phenomenological analysis to explore disclosure in adolescents with turner syndrome. British Journal of Health Psychology, 27(3), 990–1010. 10.1111/bjhp.12586 35156277 PMC9545481

[jgc470061-bib-0046] Nisselle, A. , Terrill, B. , Janinski, M. , Metcalfe, S. , & Gaff, C. (2024). Ensuring best practice in genomics education: A scoping review of genomics education needs assessments and evaluations. The American Journal of Human Genetics, 111(8), 1508–1523. 10.1016/j.ajhg.2024.06.005 38959884 PMC11339611

[jgc470061-bib-0047] Oliveri, S. , & Pravettoni, G. (2018). Capturing how individuals perceive genetic risk information: A phenomenological perspective. Journal of Risk Research, 21(2), 259–267. 10.1080/13669877.2017.1281333

[jgc470061-bib-0048] Ormond, K. E. , Hayward, L. , Wessels, T.‐M. , Patch, C. , & Weil, J. (2024). International genetic counseling: What do genetic counselors actually do? Journal of Genetic Counseling, 33, 381–391. 10.1002/jgc4.1735 37296526

[jgc470061-bib-0049] Pitney, W. , Parker, J. , Mazerolle, S. , & Potteiger, K. (2024). Qualitative research in the health professions. Taylor & Francis.

[jgc470061-bib-0050] Quinn, E. , & Mazur, K. (2022). The experiences of UK‐based genetic counsellors working in mainstream settings. European Journal of Human Genetics, 30(11), 1283–1287. 10.1038/s41431-022-01158-y 35918538 PMC9343813

[jgc470061-bib-0051] Rehm, H. L. (2017). Evolving health care through personal genomics. Nature Reviews. Genetics, 18(4), 259–267. 10.1038/nrg.2016.162 PMC6517837 28138143

[jgc470061-bib-0052] Rettie, H. , & Daniels, J. (2021). Coping and tolerance of uncertainty: Predictors and mediators of mental health during the COVID‐19 pandemic. American Psychologist, 76(3), 427–437. 10.1037/amp0000710 32744841

[jgc470061-bib-0053] Riess, O. , Sturm, M. , Menden, B. , Liebmann, A. , Demidov, G. , Witt, D. , Casadei, N. , Admard, J. , Schütz, L. , Ossowski, S. , Taylor, S. , Schaffer, S. , Schroeder, C. , Dufke, A. , & Haack, T. (2024). Genomes in clinical care. NPJ Genomic Medicine, 9(1), 20. 10.1038/s41525-024-00402-2 38485733 PMC10940576

[jgc470061-bib-0054] Rutakumwa, R. , de Vries, J. , Parker, M. , Tindana, P. , Mweemba, O. , & Seeley, J. (2020). What constitutes good ethical practice in genomic research in Africa? Perspectives of participants in a genomic research study in Uganda. Global Bioethics, 31, 169–183. 10.1080/11287462.2019.1592867 PMC7734109 33343191

[jgc470061-bib-0055] Scheinberg, T. , Young, A. , Woo, H. , Goodwin, A. , Mahon, K. L. , & Horvath, L. G. (2021). Mainstream consent programs for genetic counseling in cancer patients: A systematic review. Asia‐Pacific Journal of Clinical Oncology, 17(3), 163–177. 10.1111/ajco.13334 32309911

[jgc470061-bib-0056] Schmidlen, T. , Jones, C. L. , Campbell‐Salome, G. , McCormick, C. Z. , Vanenkevort, E. , & Sturm, A. C. (2022). Use of a chatbot to increase uptake of cascade genetic testing. Journal of Genetic Counseling, 31(5), 1219–1230. 10.1002/jgc4.1592 35616645

[jgc470061-bib-0057] Schupmann, W. , Jamal, L. , & Berkman, B. E. (2020). Re‐examining the ethics of genetic counselling in the genomic era. Journal of Bioethical Inquiry, 17(3), 325–335. 10.1007/s11673-020-09983-w 32557217 PMC10084396

[jgc470061-bib-0058] Schweizer, S. , Lawson, R. P. , & Blakemore, S.‐J. (2023). Uncertainty as a driver of the youth mental health crisis. Current Opinion in Psychology, 53, 101657. 10.1016/j.copsyc.2023.101657 37517166

[jgc470061-bib-0059] Senior, V. , Smith, J. A. , Michie, S. , & Marteau, T. M. (2002). Making sense of risk: An interpretative phenomenological analysis of vulnerability to heart disease. Journal of Health Psychology, 7(2), 157–168. 10.1177/1359105302007002455 22114235

[jgc470061-bib-0060] Sexton, A. , & James, P. A. (2022). Metaphors and why these are important in all aspects of genetic counseling. Journal of Genetic Counseling, 31(1), 34–40. 10.1002/jgc4.1463 34233383

[jgc470061-bib-0502] Shipman, H. , Flynn, S. , MacDonald‐Smith, C. F. , Brenton, J. , Crawford, R. , Tischkowitz, M. , Hulbert‐Williams, N. J. , & GTEOC Study Grp . (2017). Universal BRCA1/BRCA2 testing for ovarian cancer patients is welcomed, but with care: How women and staff contextualize experiences of expanded access. Journal of Genetic Counseling, 26(6), 1280–1291. 10.1007/s10897-017-0108-5 28540621

[jgc470061-bib-0501] Singer, C. F. , Balmaña, J. , Bürki, N. , Delaloge, S. , Filieri, M. E. , Gerdes, A.‐M. , Grindedal, E. M. , Han, S. , Johansson, O. , Kaufman, B. , Krajc, M. , Loman, N. , Olah, E. , Paluch‐Shimon, S. , Plavetic, N. D. , Pohlodek, K. , Rhiem, K. , Teixeira, M. , & Evans, D. G. (2019). Genetic counselling and testing of susceptibility genes for therapeutic decision‐making in breast cancer—An European consensus statement and expert recommendations. European Journal of Cancer, 106, 54–60. 10.1016/j.ejca.2018.10.007 30471648

[jgc470061-bib-0061] Shickh, S. , Rafferty, S. A. , Clausen, M. , Kodida, R. , Mighton, C. , Panchal, S. , Lorentz, J. , Ward, T. , Watkins, N. , Elser, C. , Eisen, A. , Carroll, J. C. , Glogowski, E. , Schrader, K. A. , Lerner‐Ellis, J. , Kim, R. H. , Chitayat, D. , Shuman, C. , Bombard, Y. , … Thorpe, K. E. (2021). The role of digital tools in the delivery of genomic medicine: Enhancing patient‐centered care. Genetics in Medicine, 23(6), 1086–1094. 10.1038/s41436-021-01112-1 33654192 PMC9306004

[jgc470061-bib-0062] Slomp, C. , Morris, E. , GenCOUNSEL Study , Knoppers, B. M. , Lynd, L. D. , Dey, A. , Adam, S. , Bansback, N. , Birch, P. , Clarke, L. , Dragojlovic, N. , Friedman, J. , Lambert, D. , Pullman, D. , Virani, A. , Wasserman, W. , Zawati, M. H. , Price, M. , Elliott, A. M. , & Austin, J. (2022). The stepwise process of integrating a genetic counsellor into primary care. European Journal of Human Genetics, 30(7), 772–781. 10.1038/s41431-022-01040-x 35095102 PMC8801315

[jgc470061-bib-0063] Smith, J. A. (2004). Reflecting on the development of interpretative phenomenological analysis and its contribution to qualitative research in psychology. Qualitative Research in Psychology, 1, 39–54. 10.1191/1478088704qp004oa

[jgc470061-bib-0064] Smith, J. A. (2011). Evaluating the contribution of interpretative phenomenological analysis. Health Psychology Review, 5(1), 9–27. 10.1080/17437199.2010.510659

[jgc470061-bib-0065] Smith, J. A. , Brewer, H. M. , Eatough, V. , Stanley, C. A. , Glendinning, N. W. , & Quarrell, O. W. J. (2006). The personal experience of juvenile Huntington's disease: An interpretative phenomenological analysis of parents' accounts of the primary features of a rare genetic condition. Clinical Genetics, 69(6), 486–496. 10.1111/j.1399-0004.2006.00624.x 16712700

[jgc470061-bib-0067] Smith, J. A. , Michie, S. , Allanson, A. , & Elwy, R. (2000). Certainty and uncertainty in genetic counselling: A qualitative case study. Psychology & Health, 15(1), 1–12. 10.1080/08870440008400284 12569931

[jgc470061-bib-0068] Smith, J. A. , Michie, S. , Stephenson, M. , & Quarrell, O. (2002). Risk perception and decision‐making processes in candidates for genetic testing for Huntington's disease: An interpretative phenomenological analysis. Journal of Health Psychology, 7(2), 131–144. 10.1177/1359105302007002398 22114233

[jgc470061-bib-0069] Smith, J. A. , & Osborn, M. (2004). Interpretative phenomenological analysis. In G. M. Breakwell (Ed.), Doing social psychology research (1st ed., pp. 229–254). Wiley. 10.1002/9780470776278.ch10

[jgc470061-bib-0503] Smith, J. A. , Flowers, P. , & Larkin, M. (2022). Interpretative phenomenological analysis: Theory, method and research (2nd ed.). SAGE. https://uk.sagepub.com/en‐gb/eur/interpretative‐phenomenological‐analysis/book250130

[jgc470061-bib-0070] Smith, J. A. (Ed.). (2024). Qualitative psychology: A practical guide to research methods. SAGE Publications.

[jgc470061-bib-0071] Snir, M. , Nazareth, S. , Simmons, E. , Hayward, L. , Ashcraft, K. , Bristow, S. L. , Esplin, E. D. , & Aradhya, S. (2021). Democratizing genomics: Leveraging software to make genetics an integral part of routine care. American Journal of Medical Genetics Part C: Seminars in Medical Genetics, 187(1), 14–27. 10.1002/ajmg.c.31866 33296144

[jgc470061-bib-0072] Sperber, N. R. , Carpenter, J. S. , Cavallari, L. H. , Damschroder, J. L. , Cooper‐DeHoff, R. M. , Denny, J. C. , Ginsburg, G. S. , Guan, Y. , Horowitz, C. R. , Levy, K. D. , Levy, M. A. , Madden, E. B. , Matheny, M. E. , Pollin, T. I. , Pratt, V. M. , Rosenman, M. , Voils, C. I. , Weitzel, K. , Wilke, R. A. , … Orlando, L. A. (2017). Challenges and strategies for implementing genomic services in diverse settings: Experiences from the implementing GeNomics in pracTicE (IGNITE) network. BMC Medical Genomics, 10(1), 35. 10.1186/s12920-017-0273-2 28532511 PMC5441047

[jgc470061-bib-0073] Spiers, J. , Smith, J. A. , Ferrer‐Duch, M. , Moldovan, R. , Roche, J. , & MacLeod, R. (2020). Evaluating a genetic counseling narrative group session for people who have tested positive for the Huntington's disease expansion: An interpretative phenomenological analysis. Journal of Genetic Counseling, 29(6), 1015–1025. 10.1002/jgc4.1229 32077165

[jgc470061-bib-0074] Starr, R. A. , & Smith, J. A. (2021). Interpretative phenomenological analysis in genetics research. In eLS (pp. 1–7). John Wiley & Sons, Ltd. 10.1002/9780470015902.a0028014

[jgc470061-bib-0504] Theed, R. , Eccles, F. J. R. , & Simpson, J. (2018). Understandings of psychological difficulties in people with the Huntington's disease gene and their expectations of psychological therapy. Psychology and Psychotherapy‐Theory Research and Practice, 91(2), 216–231. 10.1111/papt.12157 28972687

[jgc470061-bib-0075] Terry, P. E. (2020). Introducing “precision health promotion”: The convergence of genomics, health education, and lived experience. American Journal of Health Promotion, 34(3), 235–237. 10.1177/0890117120903129 31996012

[jgc470061-bib-0076] Triantafyllidi, V. E. , Mavrogianni, D. , Kalampalikis, A. , Litos, M. , Roidi, S. , & Michala, L. (2022). Identification of genetic causes in Mayer‐Rokitansky‐Küster‐Hauser (MRKH) syndrome: A systematic review of the literature. Children, 9(7), 961. 10.3390/children9070961 35883945 PMC9322756

[jgc470061-bib-0077] UK Government . (2022). Genome UK: 2022 to 2025 implementation plan for England (genome UK) [policy paper]. Department of Health & Social Care. https://www.gov.uk/government/publications/genome‐uk‐2022‐to‐2025‐implementation‐plan‐for‐england/genome‐uk‐2022‐to‐2025‐implementation‐plan‐for‐england

[jgc470061-bib-0078] Van Der Giessen, J. A. M. , Ausems, M. G. E. M. , Van Riel, E. , De Jong, A. , Fransen, M. P. , & Van Dulmen, S. (2021). Development of a plain‐language guide for discussing breast cancer genetic counseling and testing with patients with limited health literacy. Supportive Care in Cancer, 29(6), 2895–2905. 10.1007/s00520-020-05800-7 33001269 PMC8062319

[jgc470061-bib-0079] van der Schoot, V. , van der Meer, E. , Hillen, M. A. , Yntema, H. G. , Brunner, H. G. , & Oerlemans, A. J. M. (2024). Exploring uncertainties regarding unsolicited findings in genetic testing. Patient Education and Counseling, 119, 108064. 10.1016/j.pec.2023.108064 37976670

[jgc470061-bib-0080] Wagener, A. E. (2017). Metaphor in professional counseling. The Professional Counselor, 7(2), 144–154. 10.15241/aew.7.2.144

[jgc470061-bib-0081] Wainstein, T. , Elliott, A. M. , & Austin, J. C. (2023). Considerations for the use of qualitative methodologies in genetic counseling research. Journal of Genetic Counseling, 32(2), 300–314. 10.1002/jgc4.1644 36271905

[jgc470061-bib-0082] Walsh, N. , Cooper, A. , Dockery, A. , & O'Byrne, J. J. (2024). Variant reclassification and clinical implications. Journal of Medical Genetics, 61(3), 207–211. 10.1136/jmg-2023-109488 38296635

[jgc470061-bib-0083] Wieringa, G. , Dale, M. , & Eccles, F. J. R. (2022). The experience of a sample of individuals in the United Kingdom living in the pre‐manifest stage of Huntington's disease: An interpretative phenomenological analysis. Journal of Genetic Counseling, 31(2), 375–387. 10.1002/jgc4.1497 34374465

[jgc470061-bib-0084] Willig, C. (2017). Discourse analysis and Health Psychology. In Critical health psychology (pp. 199–217). Bloomsbury Publishing.

[jgc470061-bib-0085] Young, J. L. , Mak, J. , Stanley, T. , Bass, M. , Cho, M. K. , & Tabor, H. K. (2021). Genetic counseling and testing for Asian Americans: A systematic review. Genetics in Medicine, 23(8), 1424–1437. 10.1038/s41436-021-01169-y 33972720 PMC11973605

[jgc470061-bib-0086] Zakaria, W. N. A. , Yoon, S.‐Y. , Wijaya, A. , Ahmad, A. H. , Zakaria, R. , & Othman, Z. (2023). Global trends and themes in genetic counseling research. European Journal of Human Genetics, 31(10), 1181–1184. 10.1038/s41431-023-01371-3 37142766 PMC10157559

[jgc470061-bib-0087] Zhong, A. , Darren, B. , Loiseau, B. , He, L. Q. B. , Chang, T. , Hill, J. , & Dimaras, H. (2021). Ethical, social, and cultural issues related to clinical genetic testing and counseling in low‐ and middle‐income countries: A systematic review. Genetics in Medicine, 23(12), 2270–2280. 10.1038/s41436-018-0090-9 30072741

[jgc470061-bib-0088] Zierhut, H. , Betterman, S. , Kocher, M. , Tsegai, H. , & Mills, R. (2024). The state of National Institute of Health awards for funding genetic counseling research, resources, and training over the past decade. Journal of Genetic Counseling, 33, 1159–1175. 10.1002/jgc4.1850 38264803 PMC11632580

[jgc470061-bib-0089] Zybarth, D. , Inhestern, L. , Otto, R. , & Bergelt, C. (2024). Uncertainties of healthcare professionals and informal caregivers in rare diseases: A systematic review. Heliyon, 10(19), e38677. 10.1016/j.heliyon.2024.e38677 39403533 PMC11471567

